# Mug20, a novel protein associated with linear elements in fission yeast meiosis

**DOI:** 10.1007/s00294-012-0369-3

**Published:** 2012-02-24

**Authors:** Anna Estreicher, Alexander Lorenz, Josef Loidl

**Affiliations:** 1 Department of Chromosome Biology, Center for Molecular Biology of the University of Vienna (MFPL), Dr. Bohr Gasse 1, 1030 Vienna, Austria; 2 Department of Biochemistry, University of Oxford, South Parks Road, Oxford, OX1 3QU UK

**Keywords:** Meiosis, Chromosome pairing, Synaptonemal complex, Recombination

## Abstract

In the fission yeast, *Schizosaccharomyces pombe*, homologous chromosomes efficiently pair and recombine during meiotic prophase without forming a canonical synaptonemal complex (SC). Instead, it features simpler filamentous structures, the so-called linear elements (LinEs), which bear some resemblance to the axial/lateral element subunits of the SC. LinEs are required for wild-type recombination frequency. Here, we recognized Mug20, the product of a meiotically upregulated gene, as a LinE-associated protein. GFP-tagged Mug20 and anti-Mug20 antibody co-localized completely with Rec10, one of the major constituents of LinEs. In the absence of Mug20, LinEs failed to elongate beyond their initial state of nuclear dots. Foci of recombination protein Rad51 and genetic recombination were reduced. Since meiotic DNA double-strand breaks (DSBs), which initiate recombination, are induced at sites of preformed LinEs, we suggest that reduced recombination is a consequence of incomplete LinE extension. Therefore, we propose that Mug20 is required to extend LinEs from their sites of origin and thereby to increase DSB proficient regions on chromosomes.

## Introduction

Meiotic pairing of homologous parental chromosomes is imperative for crossover and the formation of chiasmata which, in turn, ensure the faithful disjunction and the reduction of the diploid somatic to haploid gametic chromosome sets. In most organisms, the formation of the synaptonemal complex (SC) is instrumental in reinforcing pairing and ensuring a proper crossover outcome of the molecular recombination process (see Page and Hawley 2004; Kleckner 2006). The SC is an elaborate structure made of longitudinal axial elements along paired chromosomes and connections (the transversal filaments) between these axes. The ultrastructure of the SC, but not the proteins of which it consists, is evolutionarily widely conserved (Bogdanov et al. 2007).

The fission yeast, *Schizosaccharomyces pombe*, is unusual in that it forms structures resembling fragmentary axial elements, while it lacks the transversal filaments between pairing chromosomes (for a review see Loidl 2006). A major component of these so-called linear elements (LinEs) is Rec10, a distant homolog of budding yeast axial element protein Red1 (Lorenz et al. 2004). Hop1 and Mek1, which are homologs of the eponymous budding yeast proteins, localize to LinEs (Lorenz et al. 2004). Therefore, the LinEs are considered evolutionary relics of the SC’s axial elements (Loidl 2006). In addition, LinEs consist of Rec25 and Rec27, which are not related to known SC proteins (Davis et al. 2008), and the SUMO protein Pmt3 has been found associated with them (Spirek et al. 2010). While Rec10, Rec25 and Rec27, which probably form a complex, are the indispensable core components for LinE formation, LinE development is only mildly affected in the absence of Hop1, Mek1 and Pmt3 (Lorenz et al. 2006; Davis et al. 2008; Spirek et al. 2010). Mutants with defective LinE formation display a strongly reduced capability of DSB formation (Cervantes et al. 2000) and genetic recombination (Wells et al. 2006). This is directly related to the requirement of LinEs for recruiting two factors, Rec7 and Rec24, to chromatin (Lorenz et al. 2006; Bonfils et al. 2011). They enable Rec12 (Spo11) to induce meiotic DNA double-strand breaks (DSBs). Homologous pairing is also reduced in the absence of LinEs (Davis et al. 2008; Molnar et al. 2003).

In a previous study, proteins interacting with the LinE component Rec10 were isolated by co-precipitation with TAP-tagged Rec10. It detected Pli1, a SUMO ligase, suggesting that a component of LinEs, most probably Rec10, becomes SUMOylated and that this contributes to full LinE functionality (Spirek et al. 2010). Among Rec10’s partners, there was also a 19 kDa protein, Mug20 (Spirek et al. 2010). Originally, the meiotically upregulated gene *mug20*, which is encoded by ORF SPBC36B7.06c, had been identified in a search for genes that are transcriptionally upregulated during meiosis (Mata et al. 2002). Deletion studies failed to detect a severe spore viability or segregation defect (Martín-Castellanos et al. 2005; Gregan et al. 2005). Here, we study the localization of the Mug20 protein and detect cytological and recombination anomalies caused by the lack of *mug20*.

## Materials and methods

### Culture and sporulation

Strains were grown at 30°C under standard conditions (see Gutz et al. 1974; Forsburg and Rhind 2006). Azygotic meiosis of diploids in liquid cultures was induced as described by Loidl and Lorenz (2009). For a list of strains see Table 1.

**Table 1:** Strain list

AEP516	*h* ^+^ *ade6*-*M210 ura4*-*D18 mug20* ^+^::*GFP*-*kanMX6*
AEP517	*h* ^−^ *ade6*-*M216 ura4*-*D18 mug20* ^+^::*GFP*-*kanMX6*
AEP540	*h* ^+^ *ade6*-*M216 leu1*-*32 rec10*-*155*::*LEU2* ^+^ *mug20* ^+^::*GFP*-*kanMX6*
AEP541	*h* ^−^ *ade6*-*M210 ura4*-*D18 leu1*-*32 rec10*Δ-*155*::*LEU2* ^+^ *mug20* ^+^::*GFP*-*kanMX6*
AEP543	*h* ^−^ *ade6*-*M210 rec10*Δ-*175*::*kanMX6 mug20* ^+^::*GFP*-*kanMX6*
AEP544	*h* ^+^ *ade6*-*M216 rec10*Δ-*175*::*kanMX6 mug20* ^+^::*GFP*-*kanMX6*
AEP550	*h* ^+^ *ade6*-*M210 ura4*-*D18 rec8*Δ::*ura4* ^+^ *mug20* ^+^::*GFP*-*kanMX6*
AEP551	*h* ^−^ *ade6*-*M216 ura4*-*D18 rec8*Δ::*ura4* ^+^ *mug20* ^+^::*GFP*-*kanMX6*
AEP553	*h* ^+^ *ade6*-*M210 ura4*-*D18 rec12*Δ::*ura4* ^+^ *mug20* ^+^::*GFP*-*kanMX6*
AEP554	*h* ^−^ *ade6*-*M216 ura4*-*D18 rec12*Δ::*ura4* ^+^ *mug20* ^+^::*GFP*-*kanMX6*
MCW1196^a^	*h* ^+^ *ade6*-*469 his3* ^+^-*aim ura4*-*D18 leu1*-*32 his3*-*D1*
MCW1197^a^	*h* ^−^ *ade6*-*M26 ura4* ^+^-*aim2 ura4*-*D18 leu1*-*32 his3*-*D1*
ALP1491	*h* ^+^ *ade6*-*469 his3* ^+^-*aim ura4*-*D18 leu1*-*32 his3*-*D1 mug20*Δ::*natMX6*
AEP556	*h* ^+^ *ade6*-*469 his3* ^+^-*aim ura4*-*D18 leu1*-*32 his3*-*D1 mug20*Δ::*natMX6*
AEP557	*h* ^−^ *ade6*-*M26 ura4* ^+^-*aim2 ura4*-*D18 leu1*-*32 his3*-*D1 mug20*Δ::*natMX6*
AEP558	*h* ^−^ *arg1*-*14 mug20*Δ::*natMX6*
AEP559	*h* ^+^ *ade6*-*M375 his4*-*239 mug20*Δ::*natMX6*

Strains were created for this study, unless noted otherwise

^a^ Osman et al. (2003)

To assay sporulation efficiency, diploid strains were grown in liquid rich medium lacking adenine. Cells were then transferred to sporulation medium and incubated for 24–36 h. Aliquots were taken and fixed in 70% ice-cold ethanol, and cells were scored for the presence of four spores or a single nucleus in DAPI-stained slides. For evaluating spore viability, haploid cells were crossed and incubated on malt extract agar until asci containing spores had formed. Unsporulated cells were removed by β-glucuronidase digestion, and a defined number of spores was plated onto rich medium. Colonies grown after 5 days were counted and the ratio of colonies to plated spores was determined.

### *mug20* knockout

For the construction of diploid *mug20* deletion strains, haploid strains of opposite mating types carrying complementing *ade6*-*M210* and *ade6*-*M216* alleles were transformed with a linearized knockout plasmid (Gregan et al. 2005), kindly provided by Juraj Gregan (see http://mendel.imp.univie.ac.at/Pombe/ for the design of the construct and the knockout procedure). Transformation resulted in the replacement of a region from 171 bp upstream to 107 bp downstream of the ORF SPBC36B7.06c with a nourseothricin resistance gene. Single colonies from clonNAT plates were screened by colony PCR for correct integration of the knockout cassette. Diploid mutant strains were produced by mating and maintained by growth in medium lacking adenine.

### Mug20 tagging and antibody construction

Mug20-GFP was created by fusing the *GFP* gene to the C terminus of the *mug20* ORF. A ca. 500 bp C-terminal fragment (excluding the stop codon) of the gene was PCR-amplified from genomic DNA using primers for creating flanking unique ApaI and XhoI restriction sites (5′-atatatgggcccttcaaccgtattccacc-3′ and 5′-atatatctcgagaaaattgtcgagaatagctttatg-3′; restriction sites underlined). The fragment was ligated to a GFP plasmid (Lindner et al. 2002) carrying a G418 resistance marker. The construct was tested for correct sequence, linearized with BlpI, and used for transforming *S. pombe* strains by homologous recombination. Transformants were grown on solid selective medium, and the correct integration of the tagging construct was checked in positive candidates by colony PCR. Haploid strains of opposite mating types carrying complementing *ade6*-alleles were produced and mated to create the diploid strain. Normal progression through meiosis indicated that the tagged protein was functional.

Polyclonal anti-Mug20 antibodies were raised in a rabbit against the internal LVQHRRNSQNKLKC and the C-terminal KMIETSTHKAILDNF peptide by a commercial provider (Eurogentec, Seraing, Belgium).

### Yeast two-hybrid assay

cDNA of candidate genes was amplified by PCR using a meiotic cDNA library (kindly provided by Hiroshi Nojima, Osaka University) as template. The products were cloned and sequenced and inserted in frame in the assay vectors pGBKT7 and pGADT7, carrying the binding and activation domains (Matchmaker GAL4 Two-Hybrid System 3, Clontech, Mountain View, CA). *S. cerevisiae* strains Y187 and AH109 (Matchmaker 3) were transformed with the two plasmids, respectively. The strains carrying the different constructs were mated and a single diploid cell of each was grown to a colony. Clones were grown to saturation in rich liquid medium, and plates with rich medium and selective-Ade and -His medium were inoculated with 1:10-dilution series of cell suspensions. Growth of cultures of different cell densities on the different plates was evaluated after 2–3 days. Experiments were repeated three times, haploid strains were mated freshly for each repeat.

### Immunoblotting

Samples from meiotic cultures were taken at 1-h intervals. Cells were broken up by glass beads in a multi-beads shocker (Yasui Kikai, Osaka, Japan) and protein extracts were prepared by trichloroacetic acid precipitation (see Spirek et al. 2010). 10 μl of extracts were run on 10% SDS-PAGE gels and blotted to Hybond-P PVDF membrane (Amersham Biosciences, GE Healthcare, Pittsburgh, PA).

Mug20-GFP was detected by incubating the blot over night at 4°C with goat anti-GFP antibody (1:2,000, Rockland, Gilbertsville, PA) in TBST (20 mM Tris pH 7.5, 140 mM NaCl, 0.05% Tween 20) + 3% dry milk. Membranes were washed and incubated for 2 h with HRP-conjugated anti-goat antibody (1:5,000, Pierce, Thermo Fisher Scientific, Rockford, IL), washed, then incubated with chemiluminescent reagent (Thermo Fisher Scientific), and exposed to X-ray film. Untagged Mug20 was detected by incubating the membranes with rabbit polyclonal antibody (1:1,000) and HRP-conjugated anti-rabbit antibody (1:10,000, Dako, Glostrup, DK), followed by a chemiluminescent reaction as above.

### Cytological methods

Cells were taken from cultures 5–7 h after induction of sporulation, when LinEs of all types are most abundant in the wild type (Loidl 2006), and meiotic nuclei were prepared by a detergent-spreading method. In short, cells were spheroplasted by enzymatic digestion of cell walls, dropped onto a slide, membranes were solubilized by the addition of a detergent (Lipsol, Barloworld Scientific) and the spread nuclei were fixed by the addition of a 4% paraformaldehyde solution supplemented with 3.4% sucrose (for a detailed protocol see Loidl and Lorenz 2009). For immunofluorescence staining, slides were washed with PBS + 0.05% Triton X-100, incubated with primary antibodies, washed, incubated with secondary antibodies, washed, and mounted in antifading buffer (Vectashield, Vector Laboratories Inc., Burlingame, CA) supplemented with 0.5 μg/ml DAPI (see Loidl and Lorenz 2009). Primary antibodies were rabbit anti-Rec10 [1:200, (Lorenz et al. 2004)], chicken anti-GFP (1:1,000, Chemicon, Temacula, CA), rabbit anti-Mug20 (1:200), and monoclonal mouse anti-Rad51 (1:50, NeoMarkers, Fremont, CA). Secondary antibodies were anti-rabbit conjugated to CY3 (1:1,000, Amersham), anti-rabbit conjugated to FITC (1:200, Sigma, St. Louis, MO), anti-chicken conjugated to CY3 (1:200, Abcam, Cambridge, UK), anti-mouse conjugated to CY3 (1:400, Jackson Immuno Research, West Grove, PA), and anti-mouse conjugated to FITC (1:100, Sigma-Aldrich, St. Louis, MO).

Slides were inspected under a fluorescence microscope equipped with the appropriate filters, and separate color channels were recorded using a cooled monochrome CCD camera. Color channels were merged and assigned artificial colors using image-processing software.

### Genetic recombination assay

Haploid wild type and *mug20*Δ strains carrying auxotrophy marker alleles were crossed and sporulated, and sporulation efficiency was checked under the light microscope. Unsporulated cells were removed by β-glucuronidase digestion, and a defined number of spores was spread onto plates with rich medium. After the formation of visible colonies, cells were replica-plated onto selective media and meiotic intragenic and intergenic recombination was assessed by screening the progeny for prototrophs.

## Results and discussion

### Spore viability and genetic recombination are reduced in *mug20*Δ

Mug20, encoded by ORF SPBC36B7.06c, is a small 19.2 kD protein without any notable domain organization and homologs in other organisms. Thus, like Rec25 and Rec27 (Davis et al. 2008) it seems to be specific for fission yeast and its unconventional SC-less meiosis.

**Fig. 1:**
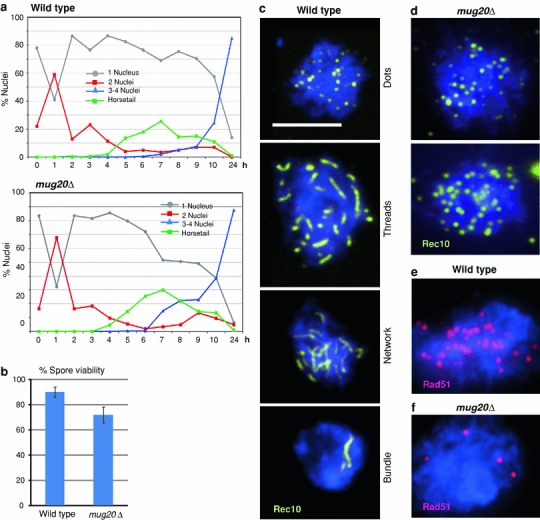
Phenotype of *mug20*Δ. **a** Comparison with a wild-type time course shows that there is little if any effect of the mutation on meiotic progression. **b** Spore viability is moderately reduced in *mug20*Δ. Mean of three experiments each, with a total of 106 and 132 tetrads scored. *Error bars* indicate standard deviations. **c** In the wild type, dots of Rec10 (*yellow*) first appear and extend to longer structures, the LinEs. In some nuclei, the LinEs form networks, and in a subset of nuclei, LinEs are concentrated in a few thick threads (which have been identified by electron microscopy as bundles of individual LinEs––Bähler et al. 1993). **d** In the mutant, LinEs do not develop beyond the dotted stage. As compared to the wild type (**e**), Rad51 foci (*red*) are markedly reduced in the mutant (**f**). Examples in **c**–**f** were taken from cultures 6 h after induction of sporulation. *Bar* 5 μm

We constructed a strain carrying a *mug20* deletion. In this strain, the progression of meiotic stages was normal with horsetail nuclei (Ding et al. 2010) and the first and second division timed normally (Fig. 1a). Sporulation efficiency was 89.1% (±7.4 SD; mean of four experiments with *n* = 1,600 cells) which did not differ notably from wild-type levels (91.8% ± 1.9; mean of four experiments with *n* = 1,600 cells). Spore viability was moderately reduced to 80% of the wild type (Fig. 1b). Inspection of 4-nucleate asci revealed that, whereas in the wild type, 96% (*n* = 104) contained four nuclei of equal sizes, in the mutant only 71% (*n* = 179) had normal nuclei and the remainder featured either four nuclei of different sizes (20%), 1–3 nuclei (4%) or a completely abnormal distribution of DNA masses.

Reduced spore viability could be a consequence of reduced or faulty recombination leading to chromosomal nondisjunction or damage. Therefore, we next tested genetic recombination, both crossing over and gene conversion, in the *mug20*Δ strain. Crossover frequencies (derived from prototroph production by intergenic recombination) were decreased in the intervals *his4*–*lys4*, *arg1*–*ade6* and *ura4* ^+^-*aim2*–*his3* ^+^-*aim*, 8.5-, 1.5- and 14.8-fold, respectively (Table 2). Gene conversion (measured as intragenic recombination frequency) rates at the *ade6* locus were reduced from 81- to 237-fold. This strong reduction was independent of the presence (*M26*) or absence (*M375*) of a hot spot, whereas at the *ura1* locus the decrease in gene conversion only amounted to 2.8-fold in the *mug20*Δ compared to wild type (Table 3). Gene conversions at the *ade6* locus within the *ura4* ^+^-*aim2*–*his3* ^+^-*aim* interval had an equal chance to be associated with a crossover in the wild type and in the mutant (Table 4). This excludes a late role in the crossover/non-crossover pathway decision for Mug20 and suggests that it acts early during meiotic recombination, possibly at the Rec12 loading or activation stage.

**Table 2:** Crossover frequency in given intervals

Interval	*his4*–*lys4*	*arg1*–*ade6*	*ura4* ^+^-*aim2*–*his3* ^+^-*aim*
Wild type	6.0 ± 0.4 (403)	14.5 ± 2.2 (903)	7.4 ± 1.0 (85)
*mug20*Δ	0.7 ± 0.3 (283)	9.7 ± 1.8 (438)	0.5 ± 0.4 (6)
*n*-Fold reduction	8.5	1.5	14.8

Percentages ± SD of prototrophic colonies formed are an average of three independent experiments. The number of prototrophic colonies evaluated is given in parentheses

**Table 3:** Gene conversion frequency at given loci

Locus	*ade6*	*ade6*	*ura1*
Cross	*ade6*-*M26* × *ade6*-*469*	*ade6*-*M375* × *ade6*-*L52*	*ura1*-*61* × *ura1*-*171*
Wild type	57 ± 2.2 (3,670)	1.7 ± 0.4 (1,289)	0.4 ± 0.1 (473)
*mug20*Δ	0.24 ± 0.13 (295)	0.021 ± 0.004 (40)	0.14 ± 0.11 (193)
*n*-Fold reduction	237	81	2.8

The number of prototrophic recombinants ± SD per 10,000 viable spores are an average of three independent experiments. The number of prototrophic colonies evaluated is given in parentheses

**Table 4:** Frequency of conversion-associated crossing over in the *ura4* ^+^-*aim2*–*ade6*–*his3* ^+^-*aim* interval

Cross (genotype)	No. of Ade^+^ colonies evaluated	Ura^−^ His^+^ (P1)	Ura^+^ His^−^ (P2)	Ura^−^ His^−^ (R1)	Ura^+^ His^+^ (R2)
MCW1196 × MCW1197 (wild type)	1,140	5.27 ± 2.66	34.75 ± 2.85	55.40 ± 5.98	4.56 ± 2.61
ALP1491 × AEP557 (*mug20*Δ)	1,217	2.33 ± 0.88	36.64 ± 4.80	59.33 ± 4.38	1.71 ± 0.93

Recombination between flanking markers was tested in cells that were Ade^+^ due to gene conversion at the *ade6* locus. Frequencies of parental (*P*) and recombinant (*R*) ± SD colonies were calculated as means from six independent crosses each. *ade6*-*M26* is a known hot spot for recombination and therefore acts predominantly as a recipient of genetic information, which explains the disparity between R1 and R2 classes. For details about the genetic markers, see Osman et al. (2003)

### LinEs do not elongate in *mug20*Δ

LinE development can be monitored by immunostaining of Rec10, one of the LinE’s major components (Fig. 1c). In the wild type, it begins as dots and seems to progress by elongation to threads and, later, their merging. Several such tracts can become laterally connected providing a reticular appearance, which is most clearly seen in the electron microscope (Bähler et al. 1993). In addition, during a later stage in LinE development, they may appear as thick rods which, in the electron microscope, have been seen to consist of bundles of LinEs (Bähler et al. 1993). In the *mug20*Δ mutant, only Rec10 dots were observed (Fig. 1d). This suggests that LinEs failed to elongate and Rec10 localization remained restricted to the initial loading sites.

The conserved strand exchange protein Rad51 associates with ssDNA during the strand invasion step in meiotic recombination (e.g., San Filippo et al. 2008) and hence it serves as a cytological marker for DSB processing. In the wild type, it localizes preferentially to LinEs (Lorenz et al. 2006). In the mutant, an average of 3.0 (SD = 1.5, *n* = 40 nuclei) Rad51 foci were counted per nucleus as compared to 22.5 (SD 6.8, *n* = 39 nuclei) foci in the wild type (Fig. 1e, f). This reduction is consistent with the observed reduction of genetic recombination and it suggests that DSB formation and/or processing is reduced in the *mug20*Δ mutant.

### Mug20 localizes to LinEs

**Fig. 2:**
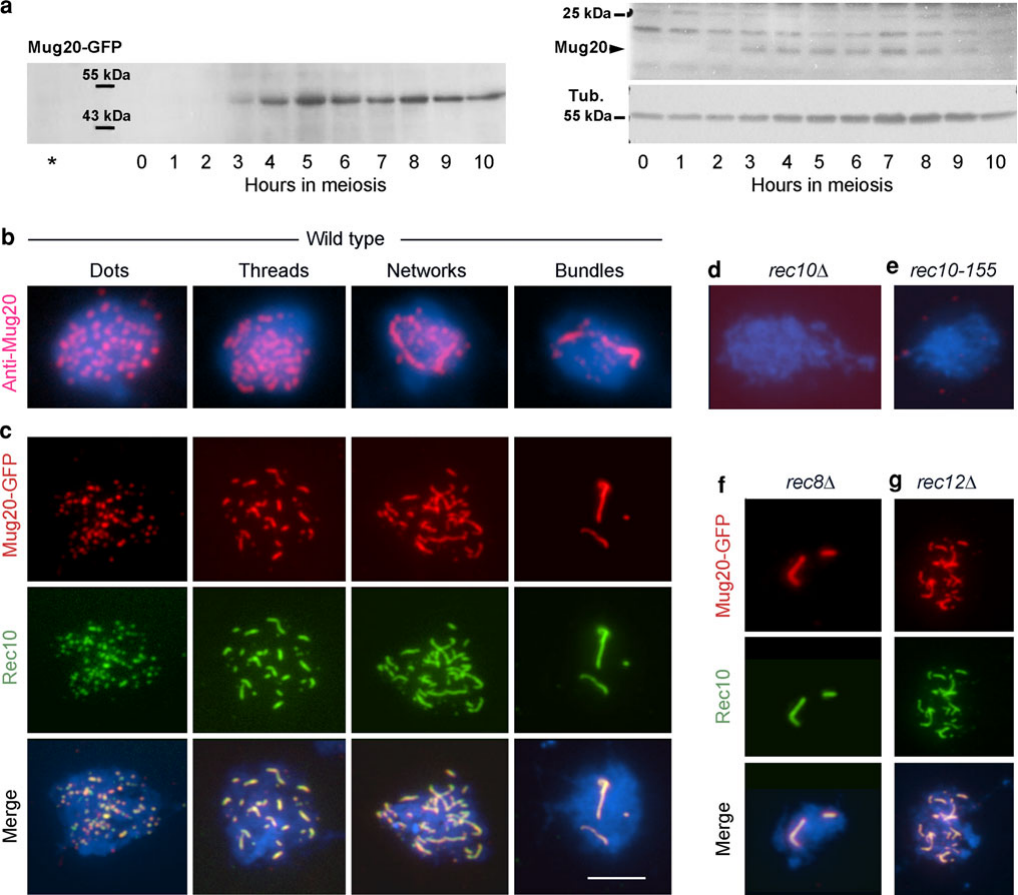
Mug20 expression and localization in meiosis. **a** Western time courses of Mug20-GFP (*left*) and Mug20 (*right*). The signals correspond to the calculated sizes of the Mug20-GFP fusion-protein (47 kDa) and Mug20 (19 kDa). The *asterisk* denotes untagged Mug20 extract from timepoint *t* = 5 h. *Tub.* α-tubulin loading marker. **b** Immunostaining of Mug20 *delineates dots and elongated structures* typical of LinEs. **c** Mug20-GFP (*red*) co-localizes with Rec10 (*green*) along LinEs at all stages. In the absence of Rec10 (**d**), and in the *rec10*-*155* mutant (**e**), Mug20 does not localize. **f** In the *rec8*Δ mutant, Mug20 highlights truncated Rec10 structures. **g** Mug20 localization to LinEs is independent of Rec12/Spo11. Examples in **b**–**g** were taken from cultures 6 h after induction of sporulation. *Bar* 5 μm

To study the expression and localization of Mug20, we constructed a GFP-tagged version, and we had antibodies produced against the protein. Immunodetection of Mug20 or Mug20-GFP on Western blots of whole cell extracts revealed that the protein was expressed from ~3 h after induction of meiosis (Fig. 2a), i.e., when LinEs begin to form (see Loidl 2006).

Cytological detection of both immunostained and GFP-tagged Mug20 demonstrated that in the wild type, Mug20 delineates structures resembling the LinEs (Fig. 2b, c). The co-localization of Mug20-GFP with Rec10 confirmed that it indeed decorates LinEs (Fig. 2c). In the absence of the LinE core component Rec10 or in the *rec10*-*155* mutant (see Wells et al. 2006), Mug20 structures were not visible, indicating that Mug20 localization depends on intact Rec10 (Fig. 2d, e). Rec8 is a meiosis-specific cohesin subunit, and in its absence only few abnormal LinEs are formed (Molnar et al. 1995, 2003; Lorenz et al. 2004). In a *rec8*Δ strain, Mug20 co-localized with Rec10 on these structures (Fig. 2f). This confirms the absolute dependency of Mug20 on the presence of Rec10.

While the reduced nuclear localization of Rad51 in the absence of Mug20 (see above) suggests a dependency of DSBs on Mug20, it is still possible that Mug20 is loaded onto LinEs in the wake of (initial) DSB formation. We tested this possibility by detecting Mug20 in a *rec12*Δ mutant. We found that Mug20 co-localizes with Rec10 LinEs in a wild-type manner, hence its loading is independent of DSBs (Fig. 2g).

**Fig. 3:**
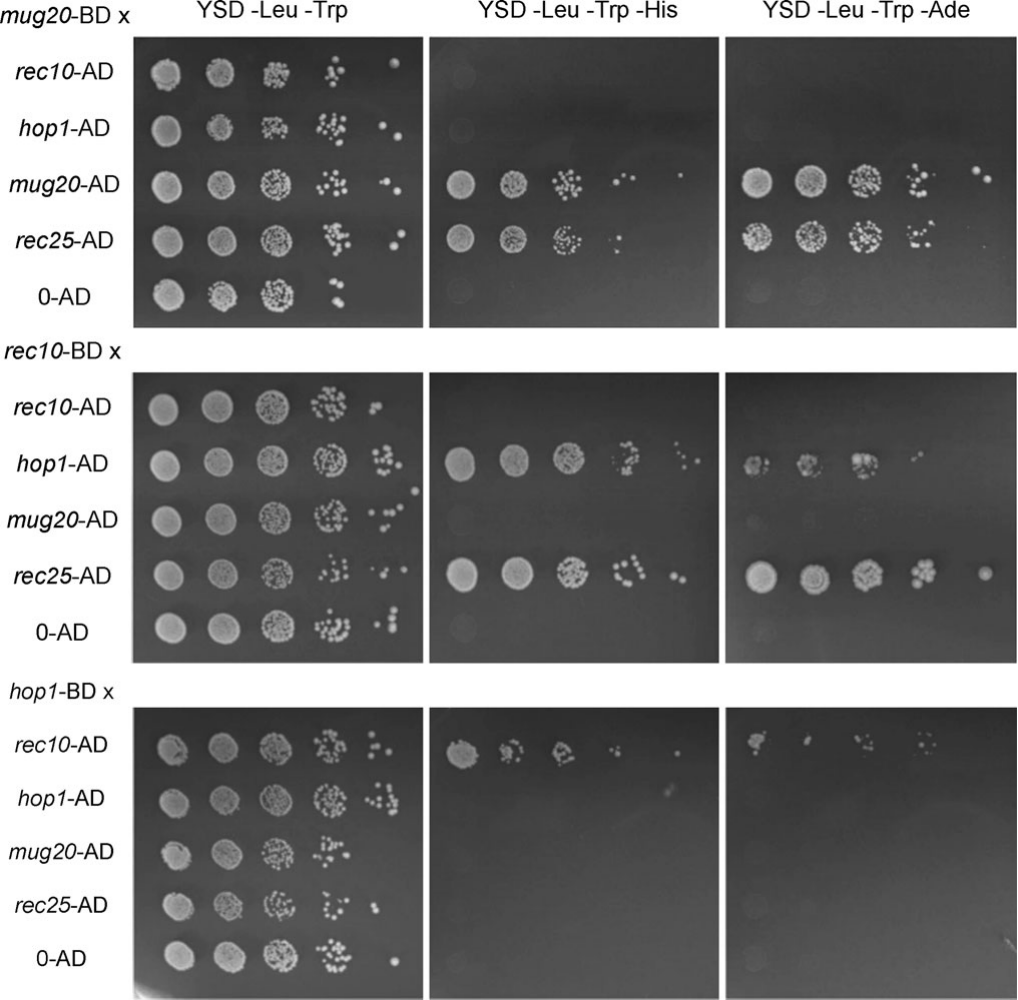
Spot tests for detecting protein-protein interactions by yeast two-hybrid assays. A budding yeast strain carrying the bait plasmid expressing Mug20 linked to the Gal4 binding domain (*BD*) was mated to strains carrying the prey plasmid expressing the Gal4 activation domain (*AD*) linked to Rec10, Hop1, Mug20 or Rec25. As a control, the bait strain was mated to a strain carrying the empty prey plasmid (0-AD). In similar experiments, Rec10 and Hop1 were used as baits. Diploid colonies were applied in a 10× dilution series to rich medium (YSD-Leu-Trp) and to selective media YSD-Leu-Trp-His and YSD-Leu-Trp-Ade. Growth on selective media indicated Mug20–Rec25 interaction and Mug20 self-interaction, and also an interaction of Rec10 with Hop1

To further corroborate the association of Mug20 with LinEs, we set up yeast two-hybrid assays for testing interactions of Mug 20 with Rec10, Rec25 and Hop1 (Fig. 3). While an interaction of Mug20 with Rec10 is evident from their co-immunoprecipitation (Spirek et al. 2010), the two-hybrid tests failed to show a direct interaction between Rec10 and Mug20. However, we found an interaction of Mug20 with Rec25 and an interaction of the latter with Rec10 (Fig. 3). Thus it is possible that Mug20 is recruited to LinEs via Rec25. From our cytological and two-hybrid data, we conclude that Mug20 localizes to a platform provided by the LinE core components Rec10 and Rec25 (and possibly Rec27).

### Functional considerations: making more of the chromosome proficient in crossing over

Foci of Rec7 and Rec24, two accessory factors for the induction of DSBs by Rec12/Spo11, are formed on LinEs (Lorenz et al. 2006; Bonfils et al. 2011). Therefore, one role of Rec10 and the other LinE core components seems to be the recruitment and/or activation of recombination factors. Surprisingly, however, a strain carrying a partial deletion mutant allele of *rec10*, *rec10*-*155*, completely fails to form LinEs and yet exhibits a reduced but still substantial level of genetic recombination (Wells et al. 2006). This suggests that the presence of a truncated version of Rec10 is sufficient for DSB formation and that this function is separable from its ability to form long complexes by auto-oligomerization. It is therefore assumed that Rec10 is required locally at the sites of recombination, and it was proposed that LinEs extend chromatin regions susceptible to DSB formation in space and time (Spirek et al. 2010).

The phenotype of *mug20*Δ resembles that of the *rec10*-*155* mutant. Since in the absence of elongated LinE tracts the available loading sites for Rec7 (and Rec24) are restricted to the initial local Rec10 assemblies, DSBs and genetic recombination are reduced. We propose that Mug20 is a factor that promotes the extension of Rec10 dots to full-sized LinEs. Its capability to self-interact in the yeast two-hybrid assay (Fig. 3) might suggest the polymerization of Mug20 as a mechanism for LinE extension. Notably, both in the *rec10*-*155* and the *mug20*Δ mutant there is a more dramatic reduction of gene conversion events than of crossovers (see above and Wells et al. 2006). This may be due to a “crossover homeostasis” and/or “crossover invariance” mechanism by which crossover levels are maintained at the expense of gene conversions under conditions of reduced DSB formation (Martini et al. 2006; Kan et al. 2011; Hyppa and Smith 2010).
